# Systematic Evolution and Study of UAGN Decoding tRNAs in a Genomically Recoded Bacteria

**DOI:** 10.1038/srep21898

**Published:** 2016-02-24

**Authors:** Nanxi Wang, Xin Shang, Ronald Cerny, Wei Niu, Jiantao Guo

**Affiliations:** 1 Department of Chemistry, University of Nebraska-Lincoln, Lincoln, Nebraska, 68588, United States; 2 Department of Chemical & Biomolecular Engineering, University of Nebraska-Lincoln, Lincoln, Nebraska, 68588, United States

## Abstract

We report the first systematic evolution and study of tRNA variants that are able to read a set of UAGN (N = A, G, U, C) codons in a genomically recoded *E. coli* strain that lacks any endogenous in-frame UAGN sequences and release factor 1. Through randomizing bases in anticodon stem-loop followed by a functional selection, we identified tRNA mutants with significantly improved UAGN decoding efficiency, which will augment the current efforts on genetic code expansion through quadruplet decoding. We found that an extended anticodon loop with an extra nucleotide was required for a detectable efficiency in UAGN decoding. We also observed that this crucial extra nucleotide was converged to a U (position 33.5) in all of the top tRNA hits no matter which UAGN codon they suppress. The insertion of U33.5 in the anticodon loop likely causes tRNA distortion and affects anticodon-codon interaction, which induces +1 frameshift in the P site of ribosome. A new model was proposed to explain the observed features of UAGN decoding. Overall, our findings elevate our understanding of the +1 frameshift mechanism and provide a useful guidance for further efforts on the genetic code expansion using a non-canonical quadruplet reading frame.

While the triplet codon is the predominant form of the current genetic code, programmed frameshifts, e.g., +1 frameshift caused by the non-canonical reading of a quadruplet codon, is a natural process. These programmed +1 frameshift events generally involve certain recoding signals that are embedded in the mRNA^1^. On the other hand, tRNA mutants that contain extended anticodon loops (8-base instead of normal 7-base loop) could induce +1 frameshift independent of recoding signals^2^,^3^,^4^,^5^,^6^,^7^,^8^,^9^. Recent developments in genetic code engineering also demonstrated that quadruplet codons could be used to encode unnatural amino acids (unAAs) under experimental conditions^10^,^11^,^12^,^13^,^14^. Here we present a systematic study on UAGN (N = A, G, U, C) decoding in order to further expand the current genetic code through suppressing quadruplet codons. Efforts have been made to expand the cellular genetic code with multiple quadruplet codons as an enabling synthetic biology tool for biological investigations^10^,^11^,^12^,^13^,^14^. Theoretically, a quadruplet codon table provides a maximum of 256 codons, which potentially allows a significant expansion of the current genetic code to facilitate biological studies^15^,^16^,^17^ and to eventually enable the ribosomal synthesis of completely artificial biopolymers as new biomaterials. Besides genetic code expansion, we also intend to use UAGN decoding as a model system to study quadruplet codon decoding (+1 frameshift) mechanism.

Two major working models were proposed to explain the mechanism of quadruplet codon decoding (+1 frameshift) with tRNAs bearing extended anticodon loops: (1) the yardstick model^18^,^19^,^20^ (Fig. 1A) states that the anticodon loop of tRNA interacts with all four bases of quadruplet codon in the A site of the ribosome, which leads to subsequent quadruplet translocation from the A site to the P site. This model is supported by observations that a number of tRNAs with an extended anticodon loop form apparent Watson-Crick complementarity to their cognate quadruplet codons at all four anticodon positions^4^,^11^,^21^,^22^,^23^,^24^,^25^. A primer extension toeprint assay on tRNA_CCCG_ also supported this theory^19^. An NMR study showed that the anticodon stem-loop (ASL) of the tRNA_CCCG_ lacked the conserved U-turn motif and could potentially undergo conformational adjustment in order to interact with quadruplet codon^19^. In an altered yardstick model^26^, the complete quadruplet codon-anticodon interaction in the A site is not required. Instead, the extra nucleotide widens the ASL and allows the anticodon nucleotide-34 to interact with either the fourth or the third and fourth codon bases. This model is supported by reported crystal structures of the 30S ribosomal subunit of *Thermus thermophilus* in complex with tRNAs known to facilitate +1 frameshifting and their cognate mRNA^26^; (2) the slippery model^7^,^27^,^28^,^29^ (Fig. 1B) entails that tRNA makes a normal three-base codon-anticodon interaction in the A site of ribosome and translocation is always triplet. An anticodon-mRNA re-pairing subsequently occurs in the P-site with a slip of the mRNA by one base, which leads to an apparent quadruplet codon decoding. The re-pairing event usually requires that the anticodon binds to a cognate or a near cognate codon in the +1 frame. For example (Fig. S9), a well-studied CCCU suppressor (tRNA^SufA6^)^5^,^27^,^30^ has a C_34_G_35_G_36_ anticodon that forms two G-C base pairs both before and after the re-pairing event, which does not cause significant penalty in binding energy. Evidences suggest that the “ribosomal grip” of the peptidyl-tRNA is pivotal for maintaining the reading frame^31^. The slippery model is also supported by observations that +1 frameshift efficiency was significantly affected by the decoding of the A-site codon following the quadruplet codon, indicating that the frameshift happens in the P site^32^,^33^,^34^,^35^. Regardless of the merits of each model, key aspects, such as the codon-anticodon interaction and translocation mechanism, of the quadruplet decoding by tRNAs with extended anticodon loops remain to be resolved.

We^14^ and other^36^ recently reported a new approach to enhance the efficiency of quadruplet codon decoding, which is based on the engineering of tRNA anticodon stem-loop (ASL). By using this method, here we present the first systematic evolution and study of tRNA variants that decode UAGN codons in a genomically recoded *E. coli* strain that lacks any endogenous in-frame UAGN sequences. Most previous studies on quadruplet codon decoding^10^,^11^,^12^,^13^,^14^ were conducted in hosts that contain a large number of in-frame four base sequences that are identical to the quadruplet codon of interest. An efficient quadruplet decoding system would lead to genome-wide, undesirable frameshifts, which could impair protein synthesis and introduce additional difficulties to the mechanistic investigations on quadruplet codon decoding. In addition, competing recognition of the first three bases of a quadruplet codon by triplet decoding tRNAs or release factors could reduce the efficiency of quadruplet codon decoding and further complicate the study. A recent synthetic biology effort led to the successful construction of an *E. coli* strain, C321.ΔA, that does not express release factor 1 (RF1; decodes the UAG nonsense codon as the termination signal of protein translation) and does not contain endogenous UAG nonsense codon (replaced with ochre UAA nonsense codon)^37^. This genomically recoded *E. coli* strain represents an excellent host for the evolution and study of UAGN decoding by eliminating the pitfalls mentioned above. In fact, a recent study^38^ showed that a *Methanocaldococcus jannaschii*-derived tRNA, 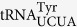, could efficiently decode UAGA codons in *E. coli* C321.ΔA by simply replacing the CUA anticodon of *M. jannaschii*-derived amber suppressor tRNA, 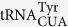, with UCUA anticodon. Here we report the engineering of the ASL of a *Methanosarcina mazei*-derived tRNA, 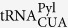, in order to efficiently decode a set of UAGN (N = A, G, U, C) codons. We chose 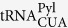 as our model tRNA since its anticodon is unlikely used as an important recognition element by its cognate PylRS according to structural data^39^. We also confirmed this notion by showing that 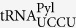, a derivative of 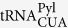 in which the anticodon CUA was replaced with UCCU, could be charged with an unAA by a PylRS variant to decode AGGA codon^14^. This structural feature allowed us to engineer the ASL of 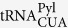 without interfering with the tRNA aminoacylation step. Our work on converting the 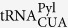 into a quadruplet codon decoding tRNA would augment the current efforts on the genetic incorporation of unAAs by PylRS variants. Since our ASL mutations in tRNA^Pyl^ unlikely affect PylRS mutants’ specificity towards their unAA substrates, evolved tRNAs can pair with PylRS mutants to incorporate a range of different unAAs.

Our ability to evolve quadruplet decoding tRNAs that are derived from the same ancestor but decode a set of UAGN codons will enable a systematic investigation on the mechanism of quadruplet codon decoding with tRNAs bearing extended anticodon loops. In addition, inferences from this work will likely bring better understanding of ribosome function in the future as well, especially how the ribosome maintains the reading frame and its relation to translocation, which remains one of the key questions to be understood in protein translation.

## Results

### tRNA (with an extended anticodon loop) library construction and selection for UAGN (N = A, G, U, C) codon decoding

An additional and randomized nucleotide, N (N = A, G, U, or C), was inserted between U33 and C34 in the anticodon loop of 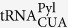. This extra nucleotide is numbered as 33.5 (Fig. 2) so that the conventional numbering of tRNA molecule at other positions was not changed. Randomization at position 33.5 resulted in a mixture of four tRNAs, including 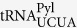-wt, 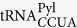-wt, 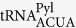-wt, and 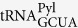-wt. The ‘wt’ designation indicates that these tRNAs have identical sequence as their parent, wild-type 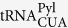 except the extra nucleotide in the anticodon loop. Each of the four tRNAs now contains an extended (eight rather than seven nucleotides) anticodon loop. We expect that such modification of tRNA can facilitate UAGN decoding since previous work suggested that tRNA mutants containing extended anticodon loops could induce +1 frameshift independent of recoding signals^2^,^3^,^4^.

As mutations in the ASL of 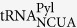 (N = A, G, U, or C) likely change the tRNA’s interaction with ribosome, mRNA, and other components of the translational machinery, we created a tRNA library (Library-NCUA11, theoretical diversity 4.2 × 10^6^; actual library size 5.2 × 10^6^) in which four nucleotides of the anticodon loop (32, 33, 37, 38; Fig. 2) and six nucleotides of the anticodon stem (29, 30, 31, 39, 40, 41; Fig. 2) were completely randomized in 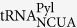. The resulting tRNA library was subsequently subjected to positive selections against UAGA, UAGG, UAGU, and UAGC codons, respectively, in *E. coli* C321.ΔA.exp in order to identify 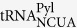 variants that can decode these quadruplet codons. Randomization of the nucleotide at position 33.5 would allow us to select 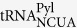 variants against fully matched or fourth-base mismatched quadruplet codons in order to examine the requirement of codon-anticodon pairing in quadruplet codon decoding. Previous data suggest that full quadruplet Watson-Crick base pairing is not absolutely required but indeed results in the highest quadruplet decoding efficiency^4^,^7^,^10^,^22^,^24^,^25^,^40^. With a systematic study and more suitable model system, we expect to gain more reliable and deeper insights into this question.

The selection was conducted in the presence of *N*ε-(tert-butyloxy-carbonyl)-L-lysine (Boc-Lys), BocLysRS (a pyrrolysyl-tRNA synthetase mutant that specifically charges 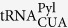 with Boc-Lys)^41^, chloramphenicol (concentrations ranging from 34 to 75 μg mL^−1^), and a chloramphenicol acetyltransferase mutant gene containing the quadruplet codon of interest at a permissive site (the codon for Gln98 is replaced with a quadruplet codon). The survivors were further screened with a range of different concentrations of chloramphenicol (50, 75, and 100 μg mL^−1^) in the presence and absence of Boc-Lys. It was considered a hit if the cell grew at 100 μg mL^−1^ chloramphenicol in the presence of Boc-Lys, but did not grow at 50 μg mL^−1^ chloramphenicol in the absence of Boc-Lys. Multiple hits were obtained for each of the UAGA, UAGG, and UAGU codons. We failed to identify any hit for UAGC codon in a number of attempts. Each hit was designated as UAGN-X, where N represents A, G, or U, and X is the hit number.

### Characterization and validation of evolved tRNAs

In order to examine the efficiency and fidelity of UAGN codon decoding by the obtained tRNAs, the four quadruplet codons of interest, UAGA, UAGG, UAGU, and UAGC were introduced into GFP_UV_ (a variant of green fluorescent protein) to replace the Asn149 codon and yielded GFP_UV_-Asn149UAGA, GFP_UV_-Asn149UAGG, GFP_UV_-Asn149UAGU, and GFP_UV_-Asn49UAGC, respectively. A series of plasmids, pGFP_UV_-NCUA-X (N = A, G, U, or C; X = hit number), were subsequently constructed. Each plasmid contains a tRNA mutant of interest (expressed under the control of a *lpp* promoter) and a GFP_UV_ mutant (driven by a T5 promoter) containing corresponding quadruplet codon. As controls, we also constructed four pGFP_UV_-NCUA-wt (N = A, G, U, or C) plasmids in which 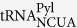-wt (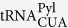 with an extra base at position 33.5) were used instead of the tRNAs mutants that were obtained from selection.

Plasmids pGFP_UV_-NCUA-X and pGFP_UV_-NCUA-wt were transformed individually into *E. coli* C321.ΔA strain containing plasmid pBK-BocLysRS that carries a copy of BocLysRS-encoding gene. Protein expression was carried out in LB medium supplemented with or without 5 mM Boc-Lys. The Fig. 3 shows the top three tRNA mutants for decoding UAGA, UAGG, and UAGU codons. All mutants exhibited significantly enhanced quadruplet decoding efficiency in the presence of Boc-Lys relative to that of the corresponding 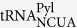-wt. The best UAGA-, UAGG-, and UAGU-decoding tRNA mutant displayed approximately 55, 40, and 9 folds improvement over their corresponding 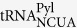-wt (Fig. 3). Notably, the decoding efficiency of UAGA and UAGG codons was much higher than that of UAGU codon. In addition, fluorescence analyses of *E. coli* cultures also showed that significant amount of full-length GFP_UV_ protein was only produced in the presence of Boc-Lys for all of our evolved 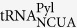 variants that were examined (Fig. 3). This result indicates that these tRNA mutants can only be charged with Boc-Lys by BocLysRS and cannot be charged with natural amino acids by BocLysRS or any endogenous aminoacyl-tRNA synthetase (aaRSs) in *E. coli*. While no hit was obtained to decode UAGC codon, we still examined the decoding efficiency of 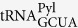-wt against UAGC codon and no detectable decoding was observed.

We further confirmed the incorporation of Boc-Lys into GFP_UV_-Asn149UAGN by mass spectrometry after SDS-PAGE separation and trypsin digest. As shown in the mass spectra (Figs S2, S3 and S4 in the Supporting Information), two mass peaks were observed in each sample. The (M + 2 H)^2+^ peak corresponds to the peptide fragment (141-LEYNYNSH**Boc-Lys**VYITADK) from GFP_UV_ that contains an intact Boc-Lys residue at position 149. The (M + 3 H)^3+^ peak corresponds to the peptide fragment that contains an lysine residue at position 149. Since the BocLysRS cannot charge 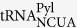 variants with lysine according to both literature report^41^ and our data (Fig. S5), the observed peptide that contains lysine at position 149 must be derived from the cleavage of the Boc group under the mass spectrometry conditions. The carbamate cleavage of Boc-Lys was also observed previously with electron spray ionization process in the literature^42^,^43^. Overall, our data confirmed that there was no undesirable incorporation of natural amino acids. The yields of the mutant GFP_UV_ proteins were 54, 9, and 21 mg/L after partial purification by affinity chromatography when tRNA mutants UAGA-1, UAGU-1, and UAGG-2 were used to decode UAGA, UAGU, and UAGG codons, respectively.

### Incorporation of other unAAs using the evolved tRNAs

Structural data shows the lack of direct interaction between PylRS and the ALS region of 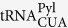. Therefore, evolved 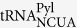 mutants in theory can be charged by PylRS mutants with unAAs other than Boc-Lys. To demonstrate the general applicability of evolved 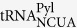 mutants, we randomly selected two PylRS variants for the incorporation of 3′-azibutyl-N-carbamoyl-lysine^44^ (AbK) and *o*-nitrobenzyl-oxycarbonyl-Nε-L-lysine^45^ (ONBK) in this study. We observed excellent selectivity when tRNA mutants were used to decode UAGN codons (AbK, Fig. S6; ONBK, Fig. S7). The relative incorporation efficiencies of Boc-Lys, Abk, and ONBK in response to either UAGN codons or UAG codon are consistent (Figs 3, S6, S7, and S8). The above results further confirmed that ASL mutations in tRNA^Pyl^ do not affect its recognition by PylRS mutants or the synthetases binding specificity towards their unAA substrates. Therefore, the evolved tRNAs can be potentially paired with any PylRS mutants for unAA incorporation, which is a significant augment of the current efforts in genetic code expansion.

### Analysis of the evolved tRNA mutants

We did not isolate and directly sequencing tRNA mutants. Instead, genes that encode the evolved tRNA mutants were sequenced. All tRNA-encoding genes were isolated and re-transformed into *E. coli* C321.ΔA for various characterizations throughout the study and consistent data were obtained. Therefore, we assume that no mutations of tRNA occurred during or after transcription and the actual tRNA sequences can be correctly deduced from their encoding genes. The observed mutations of the top three tRNAs from each of UAGA, UAGG, and UAGU selections are shown in Table 1. The most striking observation was that all of these tRNAs contained apparent UCUA (U at 33.5 position) anticodon no matter which UAGN codon they decode. In addition, some tRNAs that were selected against different UAGN codons have the same sequence, such as UAGA-2 and UAGU-3, UAGU-1 and UAGG-1, UAGU-2 and UAGG-3 (Table 1). We subsequently analysed a few less efficient tRNA variants from the selection, three, one, and one additional tRNA sequences were identified from UAGA, UAGU, and UAGG selections, respectively. As shown in Table S1, UAGU-4 and UAGG-4 have the same sequence while all UAGA hits (UAGA-4, UAGA-5, and UAGA-6) are unique. According to our data, all tRNA mutants from UAGU selection could be found from UAGA and/or UAGG selections. A large number of tRNA mutants from UAGU and UAGG selections have sequence overlap. On the other hand, most of tRNA mutants from UAGA selections are unique. Overall, the above results implicate that a fourth base-pairing between anticodon and codon is not essential for UAGN decoding.

The sequencing results also revealed three additional features of the evolved tRNA mutants: (1) no mutation was observed for the two nucleotides that are immediately 5′- or 3′-adjacent (position 33 and 37; Table 1 and Table S1) to the hypothetical four-base anticodon (NCUA). These two positions are likely essential for the function of 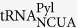-derived mutants in *E. coli*; (2) all evolved tRNA mutants contain the same A31G and U39C mutations (Table 1 and Table S1). This led to the replacement of the A31-U39 with a G31-C39 base pair, which likely strengthens the part of the anticodon stem that is directly adjacent to the anticodon loop; (3) most of the evolved tRNA mutants do not have Watson-Crick base pairing at either position 29–41 or position 30–40 (Table 1 and Table S1). These mutations likely lead to structural changes in the anticodon and ASL, which allow a favorable conformational adjustment of tRNA upon its binding to the A site and subsequent translocation to the P and E sites of the ribosome.

### Cross-decoding among UAGN codons

Three top individual tRNA mutants, UAGA-1, UAGU-1, and UAGG-2, from UAGA, UAGU, and UAGG selection, respectively, were cross-tested against codons that they were not selected against. To simplify the cross-test, a series of plasmids, pGFP_UV_-UAGN-BocLysRS (N = A, G, or U), were constructed. Each plasmid contains a BocLysRS gene (expressed under the control of a *glnS* promoter) and a GFP_UV_ mutant containing one of the UAGN (N = A, G, or U) codons. The decoding efficiencies of tRNA mutants toward each UAGN codon are correlated with the intensity of GFP fluorescence. As shown in Fig. 4, while the evolved tRNAs were among the best against the codons they were selected for, significant cross-activities were observed. Again, these results indicate that the fourth base-pairing is not essential for UAGN decoding. In addition, none of the obtained tRNA mutants could efficiently decode UAGC codon (data not shown).

### tRNA (with regular 7-base anticodon loop) library construction and selection for UAGN (N = A, G, U, C) decoding

Since the fourth base-pairing of anticodon-codon interaction is apparently not essential for UAGN decoding, we next examined if tRNA variants with regular 7-base anticodon loop could decode UAGN codons. To this end, we constructed a tRNA library (Library-CUA10, theoretical diversity 1.0 × 10^6^; actual library size 8.7 × 10^6^) based on 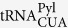 in which four nucleotides of the anticodon loop (32, 33, 37, 38; Fig. 2) and six nucleotides of the anticodon stem (29, 30, 31, 39, 40, 41; Fig. 2) were randomized. The resulting tRNA library was subsequently subjected to positive selections against UAGA, UAGG, UAGU, and UAGC, respectively, employing the same procedure as used in the Library-NCUA11 selection. No hit was obtained from these selections. The result indicates that an extra nucleotide in the anticodon loop is essential for UAGN decoding.

### Cross-decoding against UAG codons

Three top tRNA mutants, UAGA-1, UAGU-1, and UAGG-2, from UAGA, UAGU, and UAGG selection, respectively, were examined for UAG codon decoding. To do this, a plasmid, pGFP_UV_-UAG-BocLysRS, was constructed and the expression of GFP (contains an amber mutation at position 149) was examined in the presence of UAGA-1, UAGU-1, or UAGG-2. As shown in Fig. 5, UAGA-1 and UAGG-2 displayed significantly higher activity toward UAGA and UAGG codons, respectively, than to UAG codon. On the other hand, UAGU-1 displayed similar activity toward UAGU and UAG codons, which might due to the low decoding efficiency of UAGU-1 against UAGU codon. All three tRNA mutants showed similar and significantly diminished UAG decoding efficiency (~20 fold lower) than that of 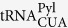.

## Discussion

This work represents the first example of a systematic evolution and study of tRNAs that decode a quartet of quadruplet codons (UAGN, N = A, U, G, or C) in a genomically recoded host with a clean background. The lack of endogenous UAG nonsense codons eliminates any in-frame UAGN sequences, which rules out the possibility of missing highly efficient UAGN-decoding tRNAs that might be cytotoxic due to undesirable reading-through of stop signals. Utilization of this host strain ensures that the most efficient UAGN-decoding tRNA variants can be identified. We targeted the evolution and study of tRNAs that are derived from the same ancestor, which enables informative and reliable comparison among obtained tRNAs. By introducing mutations into the ASL of parent tRNAs, we identified 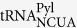 variants that displayed significant improvement in UAGN decoding efficiency in comparison to their corresponding parents. The best hit (UAGA-1) showed 55-fold improvement in UAGA decoding efficiency than that of 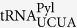-wt. Several sequence convergences were observed among the evolved tRNAs, which likely shift their interaction favorably with other components of the translational machinery for higher efficiency in quadruplet codon decoding. The observed improvement could also be partially attributed to the lack of competition from amber UAG codon-recognizing release factor-1 in the host strain. In addition to Boc-Lys, we showed that the evolved tRNA variants could be used to incorporate AbK and ONBK as well. It is likely that the evolved tRNA variants can be applied to the genetic incorporation of a range of unAAs using PylRS mutants in response to UAGN codons.

Our data suggest that an extra nucleotide in the anticodon loop of tRNA is essential for high-efficiency decoding of UAGN codons within the natural translational machinery. However, the complete four-nucleotide Watson-Crick base-pairing between anticodon and codon is apparently not required for UAGN decoding. In fact, the best tRNA hit that read UAGG and UAGU codons do not have the potential to form four-nucleotide Watson-Crick base-pairing with these codons. In addition, cross activities were observed among tRNA mutants that were selected against different UAGN codons. These results implicated that reading of quadruplet codons is less likely to happen in the A site, which is consistent with the two recent structural investigations on the ASLs of frameshift suppressor tRNAs (tRNA^SufJ^ and tRNA^SufA6^) bound to the *Thermus thermophiles* 70S A site^30^,^46^. These two structural studies suggested that +1 frameshift suppressor tRNAs do not form four-nucleotide Watson-Crick base-pairing with codons. Instead, ASL^SufJ^ and ASL^SufA6^ form a cognate and a near cognate triplet codon–anticodon interaction in the A site, respectively. It is likely that the evolved 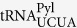 variants could engage a similar cognate or near cognate triplet interaction with the first three bases of UAGN sequences. Such cognate or a near cognate triplet codon–anticodon interactions would sufficiently lead to conformational changes of three bases, A1492, A1493, and G530, in 16S rRNA at ribosome A site^47^ and allow tRNAs to pass this critical checkpoint for decoding specificity. Since UAGN decoding does not require suppressor tRNA to occupy all four bases of the codon at the decoding center of A site, the overall and apparent quadruplet UAGN codon decoding process may be more accurately described as the induction of +1 frameshift by the evolved 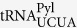 variants.

**Figure 1:**
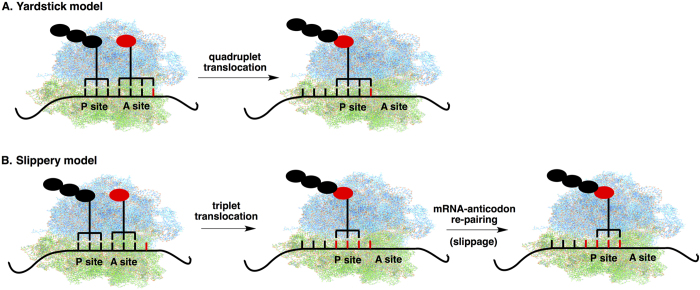
Hypothetical models for +1 frameshift (quadruplet decoding) with tRNAs containing extended anticodon loop. (**A**) The yardstick model features quadruplet anticodon-codon interaction in the A site followed by quadruplet translocation; (**B**) The Slippery model features normal triplet decoding in the A site, normal triplet translocation, and a slippage in the P site.

**Figure 2:**
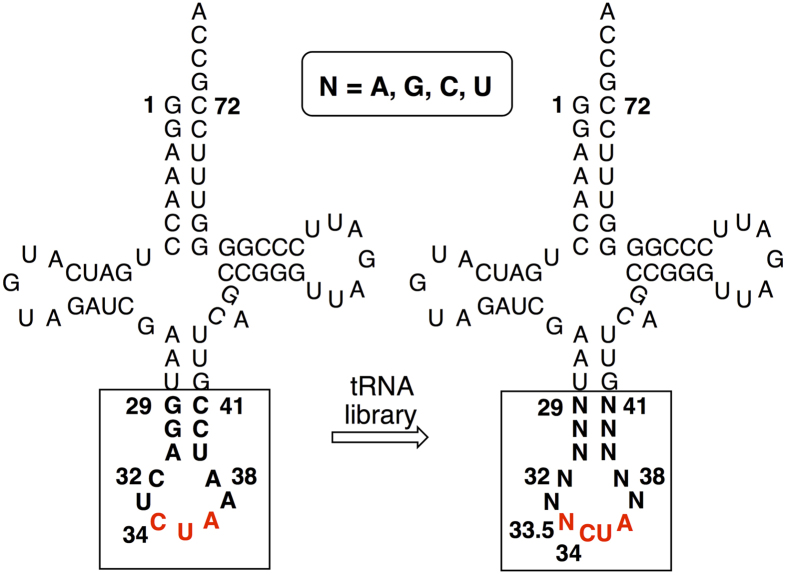
Clover leaf structures of 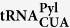 and 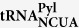. Comparing to 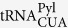, 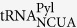 mutants contain eight rather than seven bases in its anticodon loop. The additional base, N, is numbered as 33.5. Nucleotides in the anticodon stem-loop (ASL) that were randomized are shown in bold.

**Figure 3:**
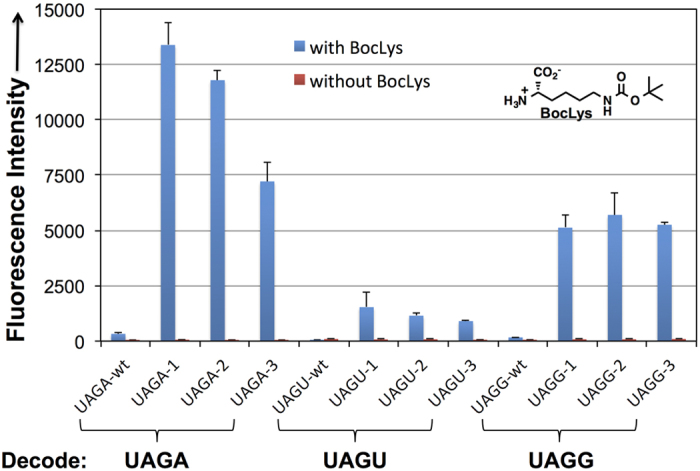
GFP_UV_ fluorescence assays of cells expressing 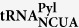 **variants.** Fluorescence readings of *E. coli* C321.ΔA cells expressing 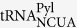-wt or the evolved 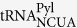 mutants, each coexpressed with BocLysRS and corresponding GFP_UV_-Asn149UAGN. The expressions were conducted either in the presence or in the absence of 5 mM Boc-Lys. Fluorescence intensity was normalized to cell growth. Each data point is the average of triplicate measurements with standard deviation.

**Figure 4:**
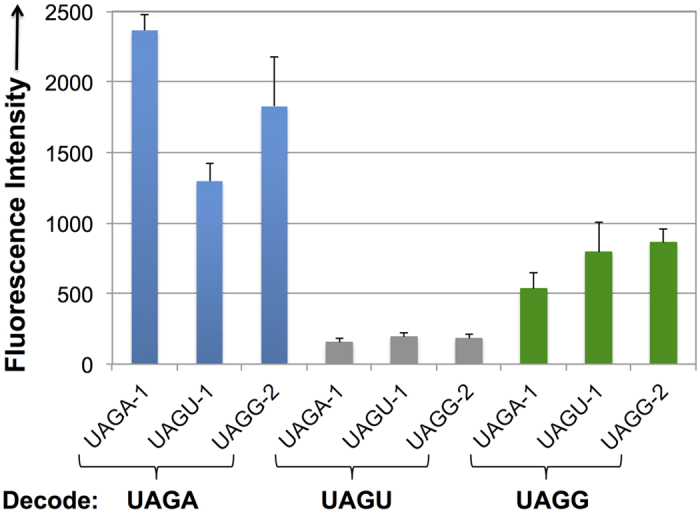
Cross-decoding among UAGN codons. Fluorescence readings of *E. coli* C321.ΔA cells expressing the GFP reporter GFP_UV_-Asn149UAGN, BocLysRS (in plasmid pGFP_UV_-UAGN-BocLysRS), and tRNA mutants (in plasmid pBK-UAGN-X; X = hit number) in the presence of 5 mM Boc-Lys. Fluorescence intensity was normalized to cell growth. Each data point is the average of triplicate measurements with standard deviation.

**Figure 5:**
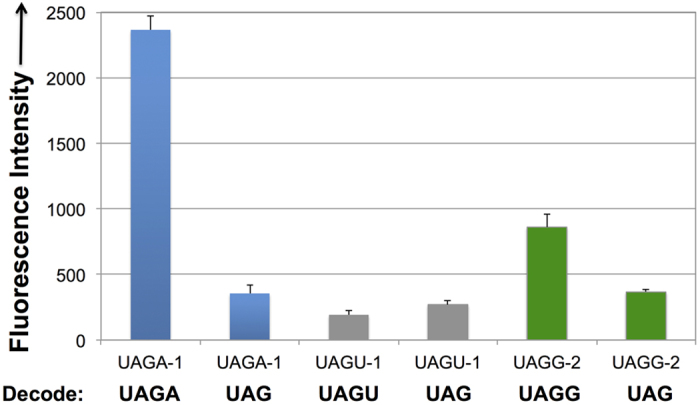
UAGN and UAG codon decoding efficiency of evolved tRNA variants. Fluorescence readings of *E. coli* C321.ΔA cells expressing the GFP reporter GFP_UV_-Asn149UAGN (or GFP_UV_-Asn149UAG), BocLysRS (in plasmid pGFP_UV_-UAGN-BocLysRS), and tRNA mutants (in plasmid pBK-UAGN-X; X = hit number) in the presence of 5 mM Boc-Lys. Fluorescence intensity was normalized to cell growth. Each data point is the average of triplicate measurements with standard deviation.

While both ours and others’ results indicate that direct quadruplet codon recognition does not happen in the A site, a question remains on how +1 frameshift happens. In the slippery model^7^,^27^,^28^,^29^, a re-pairing of tRNA to the +1 frame of mRNA (Figs 1B and S9A) occurs after the translocation of tRNA–mRNA complex from A to P site. The tRNA–mRNA re-pairing was suggested to be initiated by the P-site interaction between ribosome and the ASL of tRNA^27^. A recent structural study showed that the insertion of G37.5 in tRNA^SufA6^ destabilized the non-canonical U32–A38 base pair, which likely affected EF-G-dependent translocation and therefore facilitated +1 frameshifting^30^. The insertion of an extra nucleotide-33.5 into 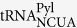 likely impaired the non-canonical C32-A38 interaction in 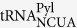 in a similar way and led to +1 frameshift. In addition, all of our tRNA hits contain either an A38U or an A38C mutation, which likely changed the finely tuned nucleotide identity of the 32–38 pair and influenced the decoding process^48^,^49^.

**Figure 6:**
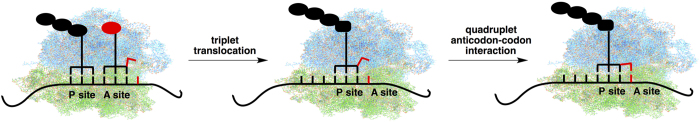
A new model for quadruplet decoding (+1 frameshift). This model features a triplet translocation followed by a quadruplet anticodon-codon interaction in the P site of ribosome.

Although our data on UAGN decoding are more consistent with the slippery model^7^,^27^,^28^,^29^, this model cannot explain why a much higher decoding efficiency was observed for UAGA codon by the evolved 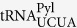 variants that have potential to form a four-nucleotide Watson-Crick base-pairing with the UAGA codon. In addition, the evolved 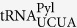 variants do not have an alternative cognate or near-cognate site for re-pairing in the P site (Fig. S9B), which is a hallmark of the slippery model. Even re-pairing would happen, we would observe that the efficiency of UAGG decoding would be higher than that of UAGA decoding since the re-pairing of the triplet anticodon of 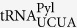 is energetically more favored for UAGG. However, we observed the opposite. Here we propose a modified slippery model for UAGN decoding (Fig. 6). The tRNA rearrangement in the P site does not necessarily involve a slippage event. It is possible for a suppressor tRNA to interact with all four bases of a quadruplet sequence in the P site and facilitate +1 frameshifting. In the cases of UAGN decoding, the extra U33.5 in 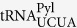 may engage certain level of interaction with the fourth base of the UAGN sequence, which creates steric interference with the incoming tRNA in the A site and induces +1 frameshift. This hypothesis is consistent with our observations on the efficiencies of UAGN suppression. The U33.5-A interaction in UAGA decoding is the most favorable one and led to the highest decoding efficiency. The U33.5-G interaction in UAGG decoding is also favorable and led to the second highest decoding efficiency. On the other hand, the U33.5-U interaction in UAGU decoding and the U33.5-C interaction in UAGC decoding are less favorable and led to the lowest decoding efficiency. In fact, we did not obtain a hit that is able to suppress UAGC codon. Other mutations in ASL of 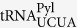 mutants may augment such interaction between tRNA and UAGN sequences by changing the conformation of engineered tRNAs. While this new model may not be suitable to explain all other +1 frameshifting cases, it provides reasonable molecular details on +1 frameshifting involving UAGN sequences.

Our current data does not pinpoint the reason for the sequence convergence to U at position 33.5 in evolved tRNAs for UAGN suppression. It is possible that only U33.5 could preserve a characteristic U-turn structure of the anticodon loop that is essential for tRNA translocation^50^,^51^. Either U33 or U33.5 interact with A37 (no mutation) or U38/C38 (A38 in the wild-type) at other side of the anticodon loop. Future structural studies would be needed to obtain more defined answers. Besides conformational factors discussed above, posttranscriptional modifications of tRNA could also play a role. The primary sequence of wild-type and evolved pyrrolysyl tRNAs may not accurately reflect the anticodon base-pairing capability for quadruplet decoding. This is because extensive and chemically diverse nucleoside modifications fine-tune the biophysical and biochemical properties of transcribed tRNA molecules^52^,^53^. In addition, certain tRNA modifications influence reading frame maintenance of the ribosome and may facilitate +1 frameshift^54^,^55^. The insertion of U33.5 may impair the normal posttranscriptional modifications of tRNA and induces reading frame shift. Currently, we are investigating the posttranscriptional modification of *M. maize* tRNA in *E. coli* host and the implication in UAGN suppression.

In summary, we have evolved a collection of tRNA mutants that are able to incorporate unAAs in response to quadruplet UAGN sequences. Our work is an augment to the current efforts in genetic code expansion. While, theoretically, a quadruplet codon table provides 256 blank codons, our data suggest that cross-recognition among quadruplet codons may significantly reduce the number of usable quadruplet codons that are intended to be used simultaneously for unAA incorporation. We are currently examining whether we are able to engineer orthogonal tRNA variants for different UAGN sequences in order to truly allow a significant expansion of the genetic code using quadruplet suppression mechanism. The present study also represents a significant step toward the elucidation of the quadruplet suppression mechanism with tRNAs bearing extended anticodon loop. Unlike most of the previous studies that only focused on limited number of +1 frameshift suppressor tRNAs, this study involves a collection of tRNAs that decode a set of UAGN codons. The inferences from this work will likely bring better understanding to how the ribosome maintains the reading frame in the future.

## Methods

### Materials and General Methods

Boc-Lys was purchased from Bachem. Primers were ordered from Sigma. Restriction enzymes, Antarctic phosphatase (AP) and T4 DNA ligase were purchased from New England Biolabs. KOD hot start DNA polymerase was purchased from EMD Millipore. Protein mass spectrometric data was collected on a Waters Synapt^®^ G2 mass spectrometer. Data was processed using Masslynx^TM^ software (Waters). Standard molecular biology techniques^56^ were used throughout. Site-directed mutagenesis was carried out using overlapping PCR. *E. coli* GeneHogs were used for routine cloning and DNA propagation. *E. coli* C321.ΔA.exp (Addgene) and C321.ΔA (Addgene) were used for quadruplet decoding tRNA selection and evaluation. All solutions were prepared in deionized water further treated by Barnstead Nanopure^®^ ultrapure water purification system (Thermo Fisher Scientific Inc). Antibiotics were added where appropriate to following final concentrations: ampicillin, 100 mg L^−1^; kanamycin, 50 mg L^−1^; tetracycline, 12.5 mg L^−1^.

### Plasmid construction

Plasmid pBK-BocLysRS was constructed by inserting BocLysRS-encoding gene behind the constitutive *glnS* promoter (*P*_*glnS*_) on pBK vector^57^.

Plasmid pREP-BocLysRS-UAGN (for positive selection) was constructed by modifying plasmid pRepCM12b^58^. Specifically, the UAG codon (position 98) in the chloramphenicol acetyltransferase-encoding gene on pRepCM12b was changed to UAGN by site-directed mutagenesis. The primers that were used for mutagenesis can be found in supporting information. The resulting plasmid, pRepCM12b-UAGN, was digested with *Xba*I and ligated to a DNA fragment containing *P*_*glnS*_-BocLysRS cassette, which was amplified from template pBK-BocLysRS, to yield plasmid pREP-BocLysRS-UAGN. Individual plasmids with four different quadruplet codons, pREP-BocLysRS-UAGA, pREP-BocLysRS-UAGG, pREP-BocLysRS-UAGU, and pREP-BocLysRS-UAGC, were isolated from single *E. coli* colonies and verified by DNA sequencing.

Plasmids pGFP_UV_-NCUA-wt (N = A, G, U, or C) were obtained by first introducing the desirable UAGN mutation into plasmid pLei-GFP_UV_-Asn149UAG^58^ by overlapping PCR. The primers that were used for mutagenesis can be found in supporting information. The resulting plasmid, pGFP_UV_-UAGN, was subsequently modified by replacing the original tRNA with 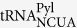-wt and yielded plasmids, pGFP_UV_-NCUA-wt (N = A, G, U, or C).

Variants of pGFP_UV_-NCUA-X (N = A, G, U, or C; X = hit number) were constructed by replacing the wild-type 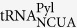-wt on pGFP_UV_-NCUA-wt with evolved tRNA mutants.

Plasmids pGFP_UV_-UAGN-BocLysRS (N = A, G, U, or C) were obtained by deleting tRNA fragment and inserting DNA fragment containing *P*_*glnS*_-BocLysRS cassette into *Pst*I and *Spe*I sites of plasmids pGFP_UV_-UCUA-wt. The *P*_*glnS*_-BocLysRS cassette was amplified from template pBK-BocLysRS. Individual plasmids, each of which contains a specific UAGN codon of interest, were isolated from single *E. coli* colonies and verified by DNA sequencing.

Plasmids pGFP_UV_-UAG-BocLysRS was obtained by deleting the tRNA fragment and inserting DNA fragment containing *P*_*glnS*_-BocLysRS cassette into *Pst*I and *Spe*I sites of plasmids pLei-GFP_UV_-Asn149UAG^58^. The *P*_*glnS*_-BocLysRS cassette was amplified from template pBK-BocLysRS.

### tRNA library construction

Procedures for library construction were adapted from Noren and Noren^59^. Mutants of tRNA were obtained by overlapping PCR using pBK-mmPylT^45^ as the template. Resulting PCR products contain DNA cassettes of *lpp* promoter-tRNA mutant-rrnC terminator. Digestion of PCR products with *Nco*I and *Xho*I followed by ligation between *Nco*I and *Xho*I sites of pBK vector resulted in the tRNA library. The primers that were used for library construction can be found in supporting information.

### Positive selection

Library DNAs were transformed into *E. coli* C321.ΔA.exp or C321.ΔA electrocompetent cells containing plasmid pREP-BocLysRS-UAGN (N = A, G, U, or C). Transformants were cultivated in LB media containing kanamycin and tetracycline. After 12 h of cultivation, cells were harvested. Based on calculation, a certain number of cells (>4.6× the size of the library) were plated on LB agar containing kanamycin, tetracycline, Boc-Lys (5 mM), and chloramphenicol (concentrations range from 34 mg L^−1^ to 75 mg L^−1^). The selection plates were incubated at 37 °C (C321.ΔA.exp) or 30 °C (C321.ΔA) for 48 h. Selected numbers of single colonies were further screened by replication onto plates with certain concentrations (50, 75, or 100 μg mL^−1^) of chloramphenicol in the presence and absence of Boc-Lys. Only the ones that grew in the presence of Boc-Lys and did not grew in the absence of Boc-Lys were selected for further evaluation.

### Fluorescence analysis of bacterial culture

*E. coli* C321.ΔA strain harboring plasmid pBK-BocLysRS and a pGFP_UV_-NCUA variant (wt or evolved tRNA mutant) was cultured in 1 mL LB media containing ampicillin, kanamycin, and chloramphenicol at 30 °C. After 18 h cultivation, 50 μL culture was sub-cultured in 1 mL LB medium containing ampicillin, kanamycin, chloramphenicol, IPTG (0.1 mM), and 5 mM Boc-Lys (or 5 mM Lys, or none). Following cultivation at 30 °C for an additional 16 h, 1 mL of cell culture were collected, washed, then resuspended in 1 mL of potassium phosphate buffer (50 mM, pH 7.4). The processed cells were directly used for fluorescence and cell density measurements using a Synergy^TM^ H1 Hybrid plate reader (BioTek Instruments). The fluorescence of GFP_UV_ was monitored at λ_Ex_ = 390 nm and λ_Em_ = 510 nm. The cell density was estimated by measuring the sample absorbance at 600 nm. Values of fluorescence intensity were normalized to cell growth. The incorporations of AbK and ONBK followed the same procedure using previously reported PylRS mutants^44^,^60^. Reported data are the average of three biological replicates with standard deviations. The cross test among different UAGN codons were conducted using the plasmid combination of pBK-tRNA-hit and pGFP_UV_-UAGN-BocLysRS.

### Protein expression and purification

*E. coli* C321.ΔA strain harboring plasmid pBK-BocLysRS and a pGFP_UV_-NCUA variant was cultured in 5 mL LB media containing kanamycin and chloramphenicol at 30 °C. After 18 hours of cultivation, 0.5 mL culture was sub-cultured in 50 mL medium. The protein expression was induced at the OD_600nm_ of 0.6 by additions of IPTG (0.5 mM) and Boc-Lys (5 mM). Cells were collected by centrifugation at 5 000 g and 4 °C for 15 min. Harvested cells were resuspended in lysis buffer containing potassium phosphate (20 mM, pH 7.4), NaCl (150 mM) and imidazole (10 mM). Cells were subsequently disrupted by sonication. Cellular debris was removed by centrifugation (21 000 g, 30 min, 4 °C). The cell-free lysate was applied to Ni Sepharose 6 Fast Flow resin (GE Healthcare). Protein purification followed manufacture's instructions. Protein concentrations were determined by Bradford assay (Bio-Rad). Purified protein was isolated by SDS-PAGE and digested with trypsin prior to MS analysis.

## Additional Information

**How to cite this article**: Wang, N. *et al*. Systematic Evolution and Study of UAGN Decoding tRNAs in a Genomically Recoded Bacteria. *Sci. Rep.* **6**, 21898; doi: 10.1038/srep21898 (2016).

## Supplementary Material

Supplementary Information

## Figures and Tables

**Table 1:** Evolved 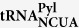 variants with improved UAGN decoding activity. Sequences of each 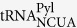 variant at randomized positions are listed.

Codon	tRNA variants	Positions				
		29–31	32,33	33.5	37, 38	39–41
UAGA	UAGA-wt	G G A	C U	U	A A	U C C
	UAGA-1	G G G	C U	U	A U	C C U
	UAGA-2	U G G	A U	U	A C	C U U
	UAGA-3	C G G	A U	U	A C	C U U
UAGU	UAGU-wt	G G A	C U	A	A A	U C C
	UAGU-1	U G G	C U	U	A U	C U U
	UAGU-2	A G G	C U	U	A U	C U U
	UAGU-3	U G G	A U	U	A C	C U U
UAGG	UAGG-wt	G G A	C U	C	A A	U C C
	UAGG-1	U G G	C U	U	A U	C U U
	UAGG-2	G G G	C U	U	A U	C U U
	UAGG-3	A G G	C U	U	A U	C U U
