# Supplementary material for: Systematic Evolution and Study of UAGN Decoding tRNAs in a Genomically Recoded Bacteria
Source: Sci Rep. 2016 Feb 24;6:21898. doi: 10.1038/srep21898 (PMC4764823; doi:10.1038/srep21898)
Supplement: Supplementary Information [file srep21898-s1.pdf]

Systematic Evolution and Study of UAGN Decoding tRNAs in a Genomically Recoded Bacteria

Nanxi Wang,[1] Xin Shang,[1] Ronald Cerny,[1] Wei Niu,*[2] Jiantao Guo*[1]

1. Department of Chemistry, University of Nebraska-Lincoln, Lincoln, Nebraska, 68588, United States.

2. Department of Chemical & Biomolecular Engineering, University of Nebraska-Lincoln, Lincoln, Nebraska, 68588, United States.

*To whom correspondence should be addressed: jguo4@unl.edu and wniu2@unl.edu

Supporting Information

Table of Contents

I. Primers for library construction....3

II. Plasmids.... 4

III. Additional data

1. Figure S1.... 5
2. Figure S2.... 6
3. Figure S3.... 7
4. Figure S4.... 8
5. Figure S5.... 9
6. Figure S6.... 10
7. Figure S7.... 11
7. Figure S8.... 12
7. Figure S9.... 13
8. Table S1.... 14

I Primers for library construction.

Library-NCUA11:

Lib-F1: CATGCCATGGGTTCCACAGGGTAGCCAGCAGC

Library-NCUA11-R1: ATTCGATCTACATGATCAGGT

Library-NCUA11-F2:

TCATGTAGATCGAAT**NNNNNN**CUA**NNNNN**GTTCAGCCGGGTTAGATTC

Lib-R2: CCGCTCGAGCAGAACATATCCATCGCGTCCGC

Library-CUA10

Lib-F1: CATGCCATGGGTTCCACAGGGTAGCCAGCAGC

Library-CUA10-R1: ATTCGATCTACATGATCAGGT

Library-CUA10-F2: TCATGTAGATCGAAT**NNNNN**CUA**NNNNN**GTTCAGCCGGGTTAGATTC

Lib-R2: CCGCTCGAGCAGAACATATCCATCGCGTCCGC

Primers for mutagenesis.

pREP-BocLysRS-UAGN:

5'-CGCTCTAGACAATTGGTGCAC-3'

5'-CTCATGGAAAACGGTGTAACAAG-3'

5'-ACCGTTTTCCATGAG**TAGN**ACTGAAACGTTTTCATCGCTCTG-3'

5'-CCACTCATCGCAGTACTGTTG-3'

pLei-GFP$_{UV}$-Asn149UAGN-tRNA-UAGN-wt (N = A, G, U, or C):

5'-GAGAAATTACATATGAGTAAAG-3'

5'-GTGTGAGTTATAGTTGTACTC-3'

5'-GTACAACTATAACTCACAC**TAGA**GTATACATCACGGCAGAC-3'

5'-GTACAACTATAACTCACAC**TAGG**GTATACATCACGGCAGAC-3'

5'-GTACAACTATAACTCACAC**TAGT**GTATACATCACGGCAGAC-3'

5'-GTACAACTATAACTCACAC**TAGC**GTATACATCACGGCAGAC-3'

5'-GATGGAGCTCTTTGTAGAGTTC-3'

II Plasmids

pBK-BocLysRS	*P_{glnS} BocLysRS, Kan^R, ColE1*
pBK-AbkRS	*P_{glnS} AbkRS, Kan^R, ColE1*
pBK-ONBKRS	*P_{glnS} ONBKRS, Kan^R, ColE1*
pREP-BocLysRS-UAGA	*P_{glnS} BocLysRS, Tet^R, Cm^R (UAGA98), p15a*
pREP-BocLysRS-UAGU	*P_{glnS} BocLysRS, Tet^R, Cm^R (UAGU98), p15a*
pREP-BocLysRS-UAGG	*P_{glnS} BocLysRS, Tet^R, Cm^R (UAGG98), p15a*
pREP-BocLysRS-UAGC	*P_{glnS} BocLysRS, Tet^R, Cm^R (UAGC98), p15a*
$pGFP_{UV}$-UCUA-wt	*T5 GFP_{UV} (Asn149UAGA), lpp $tRNA_{UCUA}$, Cm^R, p15a*
$pGFP_{UV}$-ACUA-wt	*T5 GFP_{UV} (Asn149UAGA), lpp $tRNA_{ACUA}$, CmR, p15a*
$pGFP_{UV}$-CCUA-wt	*T5 GFP_{UV} (Asn149UAGA), lpp $tRNA_{CCUA}$, Cm^R, p15a*
$pGFP_{UV}$-GCUA-wt	*T5 GFP_{UV} (Asn149UAGA), lpp $tRNA_{GCUA}$, Cm^R, p15a*
$pGFP_{UV}$-UAGN	*T5 GFP_{UV} (Asn149UAGA), Cm^R, p15a*
$pGFP_{UV}$-NCUA-X	*T5 GFP_{UV} (Asn149UAGA), lpp tRNA hit-X, Cm^R, p15a*
$pGFP_{UV}$-UAGA-BocLysRS	*T5 GFP_{UV} (Asn149UAGA), P_{glnS} BocLysRS, Cm^R, p15a*
$pGFP_{UV}$-UAGU-BocLysRS	*T5 GFP_{UV} (Asn149UAGU), P_{glnS} BocLysRS, Cm^R, p15a*
$pGFP_{UV}$-UAGG-BocLysRS	*T5 GFP_{UV} (Asn149UAGG), P_{glnS} BocLysRS, Cm^R, p15a*
$pGFP_{UV}$-UAGC-BocLysRS	*T5 GFP_{UV} (Asn149UAGC), P_{glnS} BocLysRS, Cm^R, p15a*
$pGFP_{UV}$-UAG-BocLysRS	*T5 GFP_{UV} (Asn149UAG), P_{glnS} BocLysRS, Cm^R, p15a*
pBK-library-NCUA11	*lpp $tRNA_{NCUA}$ library, Kan^R, ColE1*
pBK-Library-CUA10	*lpp $tRNA_{CUA}$ library, Kan^R, ColE1*
pBK-tRNA-hit	*lpp $tRNA_{NCUA}$ hit, Kan^R, ColE1*

III Additional data

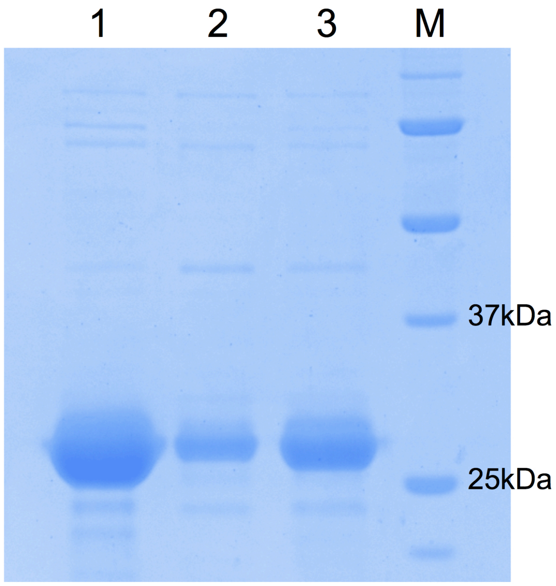

Figure S1. Purification of GFPuv mutants.

Proteins were purified using Ni Sepharose 6 Fast Flow resin. Lane 1, purified mutant GFPuv protein that was obtained from the suppression of an UAGA codon by tRNA hit UAGA-1; Lane 2, purified mutant GFPuv protein that was obtained from the suppression of an UAGU codon by tRNA hit UAGU-1; Lane 3, purified mutant GFPuv protein that was obtained from the suppression of an UAGG codon by tRNA hit UAGG-2; lane 4, molecular weight marker.

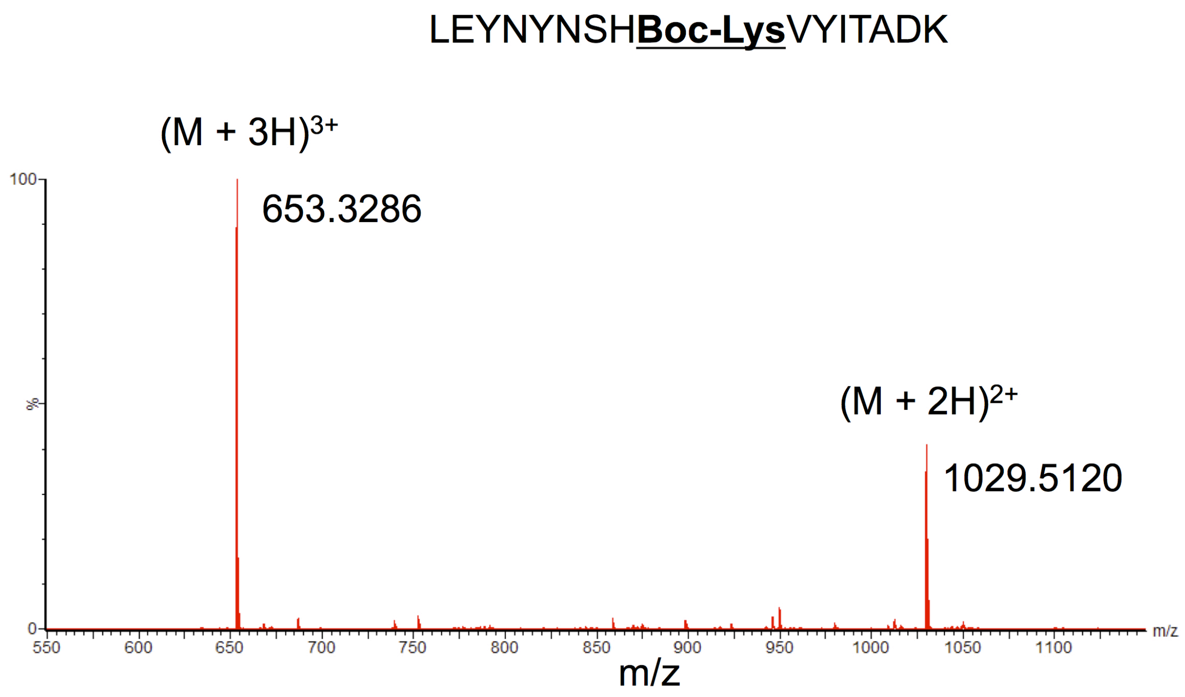

Figure S2. Mass spectrometry analysis of GFPuv containing Boc-Lys at position 149 (suppression of an UAGU codon).
The amino acid sequence of the peptide fragment, LEYNYNSH**Boc-Lys**VYITADK, from mutant GFPuv containing Boc-Lys is shown on top. Two mass speaks were observed. The $(M + 2H)^{2+}$ peak corresponds to LEYNYNSH**Boc-Lys**VYITADK that contains an intact Boc-Lys residue at position 149. The $(M + 3H)^{3+}$ peak corresponds to LEYNYNSH**K**VYITADK that contains a lysine residue at position 149 due to the loss of Boc group under the mass spectrometry conditions. (**Note**: The Boc-LysRS cannot charge the tRNA with lysine. The observed peptide that contains a lysine at position 149 must be derived from the cleavage of the Boc group.)

Theoretical monoisotopic $(M + 3H)^{3+}$ ion: 653.3272
Observed monoisotopic $(M + 3H)^{3+}$ ion: 653.3286

Theoretical monoisotopic $(M + 2H)^{2+}$ ion: 1029.5131
Observed monoisotopic $(M + 2H)^{2+}$ ion: 1029.5120

LEYNYNSH<u>**Boc-Lys**</u>VYITADK

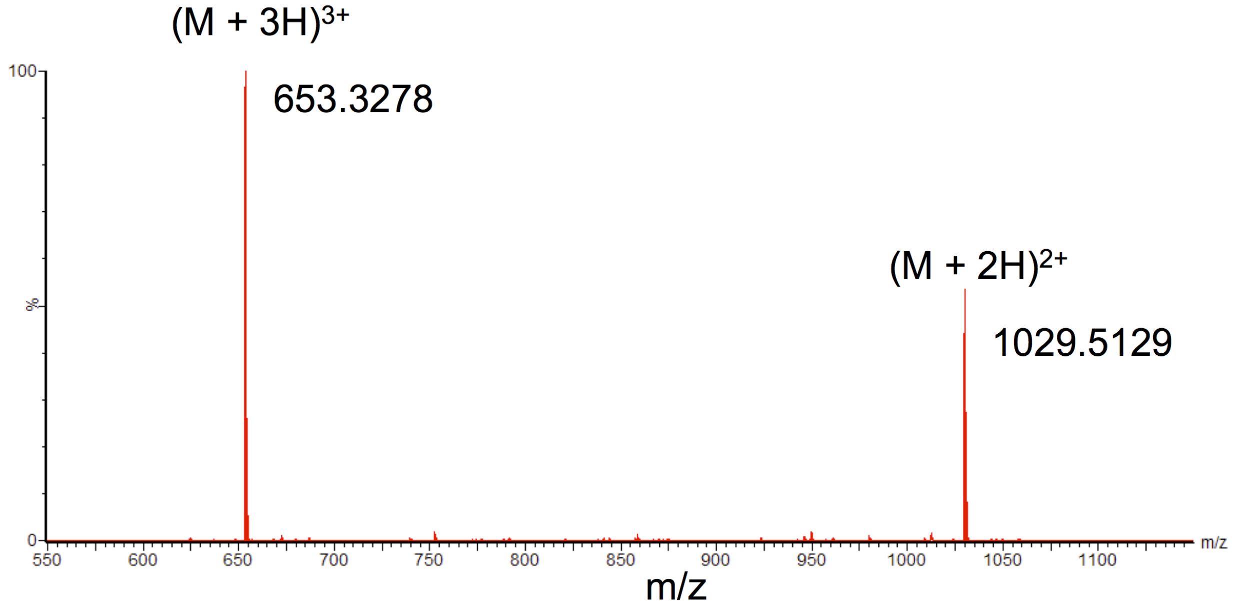

Figure S3. Mass spectrometry analysis of GFPuv containing Boc-Lys at position 149 (suppression of an UAGG codon).
The amino acid sequence of the peptide fragment, LEYNYNSH**Boc-Lys**VYITADK, from mutant GFPuv containing Boc-Lys is shown on top. Two mass speaks were observed. The $(M + 2H)^{2+}$ peak corresponds to LEYNYNSH**Boc-Lys**VYITADK that contains an intact Boc-Lys residue at position 149. The $(M + 3H)^{3+}$ peak corresponds to LEYNYNSH**K**VYITADK that contains a lysine residue at position 149 due to the loss of Boc group under the mass spectrometry conditions. (**Note**: The Boc-LysRS cannot charge the tRNA with lysine. The observed peptide that contains a lysine at position 149 must be derived from the cleavage of the Boc group.)

Theoretical monoisotopic $(M + 3H)^{3+}$ ion: 653.3272
Observed monoisotopic $(M + 3H)^{3+}$ ion: 653.3278

Theoretical monoisotopic $(M + 2H)^{2+}$ ion: 1029.5131
Observed monoisotopic $(M + 2H)^{2+}$ ion: 1029.5129

LEYNYNSH**Boc-Lys**VYITADK

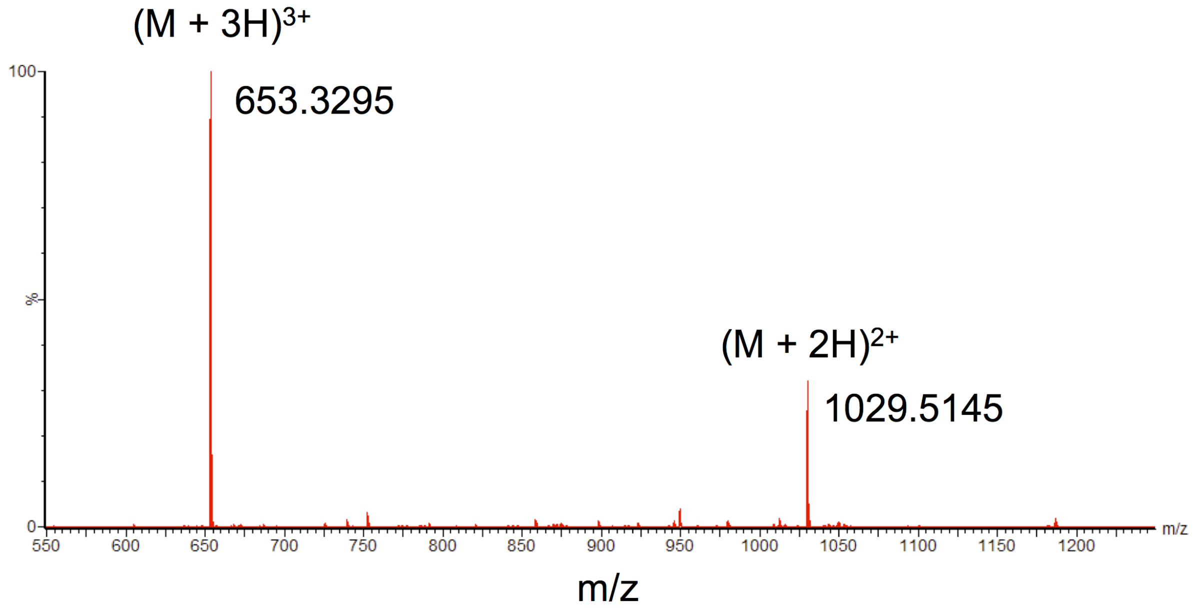

Figure S4. Mass spectrometry analysis of GFPuv containing Boc-Lys at position 149 (suppression of an UAGA codon).
The amino acid sequence of the peptide fragment, LEYNYNSH**Boc-Lys**VYITADK, from mutant GFPuv containing Boc-Lys is shown on top. Two mass speaks were observed. The $(M + 2H)^{2+}$ peak corresponds to LEYNYNSH**Boc-Lys**VYITADK that contains an intact Boc-Lys residue at position 149. The $(M + 3H)^{3+}$ peak corresponds to LEYNYNSH**K**VYITADK that contains a lysine residue at position 149 due to the loss of Boc group under the mass spectrometry conditions. (**Note**: The Boc-LysRS cannot charge the tRNA with lysine. The observed peptide that contains a lysine at position 149 must be derived from the cleavage of the Boc group.)

Theoretical monoisotopic $(M + 3H)^{3+}$ ion: 653.3272
Observed monoisotopic $(M + 3H)^{3+}$ ion: 653.3295

Theoretical monoisotopic $(M + 2H)^{2+}$ ion: 1029.5131
Observed monoisotopic $(M + 2H)^{2+}$ ion: 1029.5145

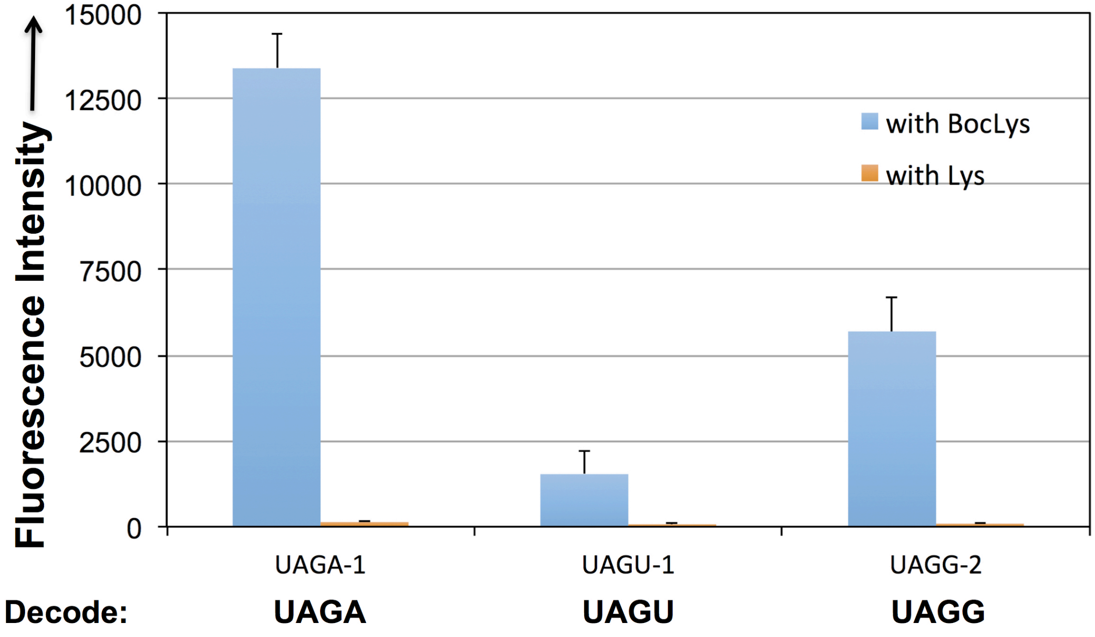

Figure S5. Genetic incorporation of Boc-Lys versus Lys.

Fluorescence readings of *E. coli* C321.ΔA cells expressing the evolved $tRNA^{Pyl}_{NCUA}$ mutants, each co-expressed with BocLysRS and corresponding GFP_{UV}-Asn149UAGN. The expression tests were conducted in the presence of either 5 mM BocLys or 5 mM Lys. The results indicated that the BocLysRS is unable to incorporate Lys and that the observed Lys residues in the mass spectrometry analyses were from the degradation of Boc-Lys. Fluorescence intensity was normalized to cell growth. Each data point is the average of triplicate measurements with standard deviation.

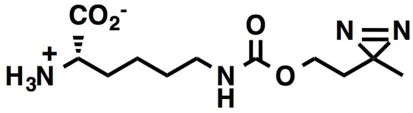

AbK

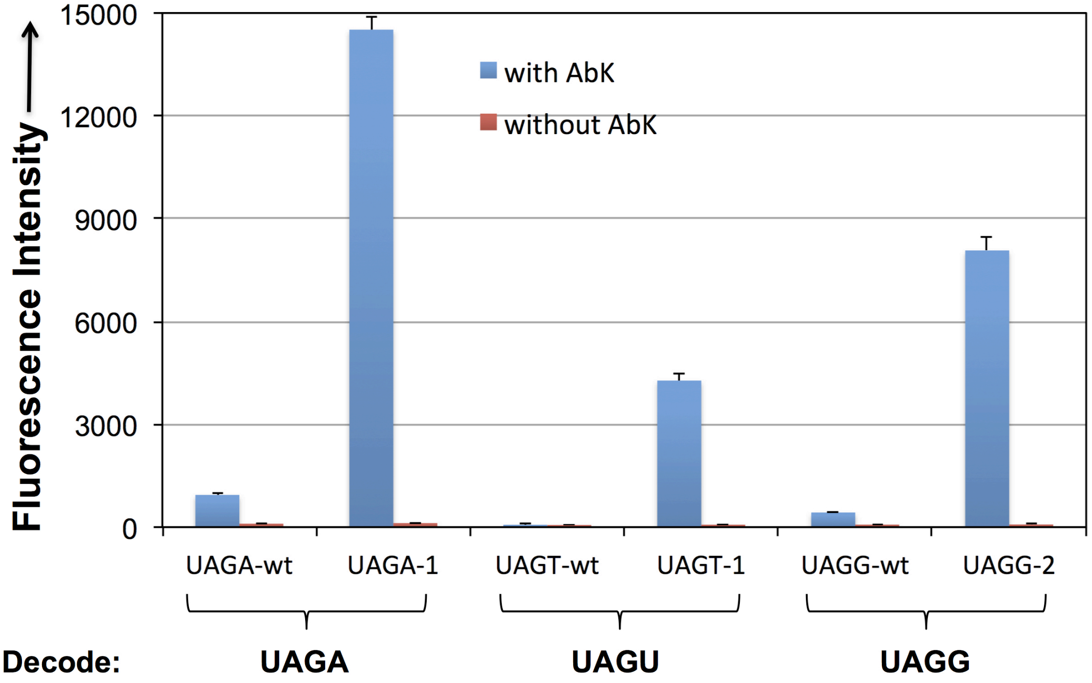

Figure S6. Incorporation of AbK using the evolved $tRNA^{Pyl}_{NCUA}$ **variants.**

Fluorescence readings of *E. coli* C321.ΔA cells expressing $tRNA^{Pyl}_{NCUA}$-wt or the evolved $tRNA^{Pyl}_{NCUA}$ mutants, each coexpressed with AbKRS (a pyrrolysyl-tRNA synthetase mutant that specifically charges $tRNA^{Pyl}$ with Abk) and corresponding GFP_{UV}-Asn149UAGN. The expressions were conducted either in the presence or in the absence of 1 mM AbK. Fluorescence intensity was normalized to cell growth. Each data point is the average of triplicate measurements with standard deviation. AbK, 3'-azibutyl-N-carbamoyl-lysine.

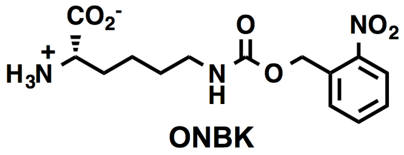

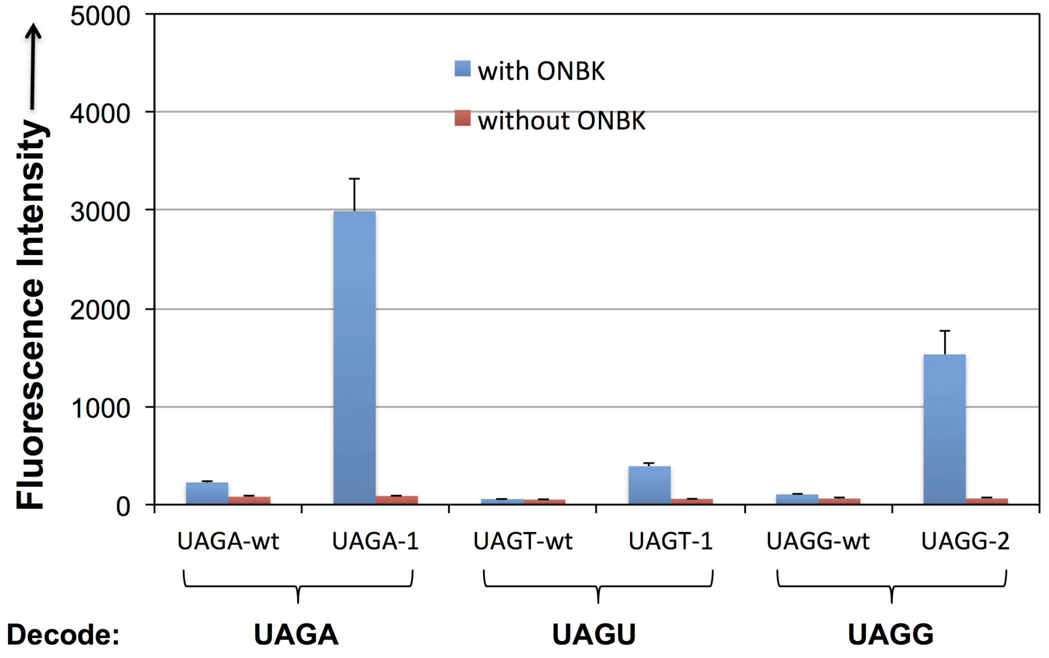

Figure S7. Incorporation of ONBK using the evolved $tRNA^{Pyl}_{NCUA}$ **variants.**

Fluorescence readings of *E. coli* C321.ΔA cells expressing $tRNA^{Pyl}_{NCUA}$-wt or the evolved $tRNA^{Pyl}_{NCUA}$ mutants, each coexpressed with ONBKRS (a pyrrolysyl-tRNA synthetase mutant that specifically charges $tRNA^{Pyl}$ with ONBK) and corresponding GFP_{UV}-Asn149UAGN. The expressions were conducted either in the presence or in the absence of 1 mM ONBK. Fluorescence intensity was normalized to cell growth. Each data point is the average of triplicate measurements with standard deviation. ONBK, *o*-nitrobenzyl-oxycarbonyl-Nε-L-lysine.

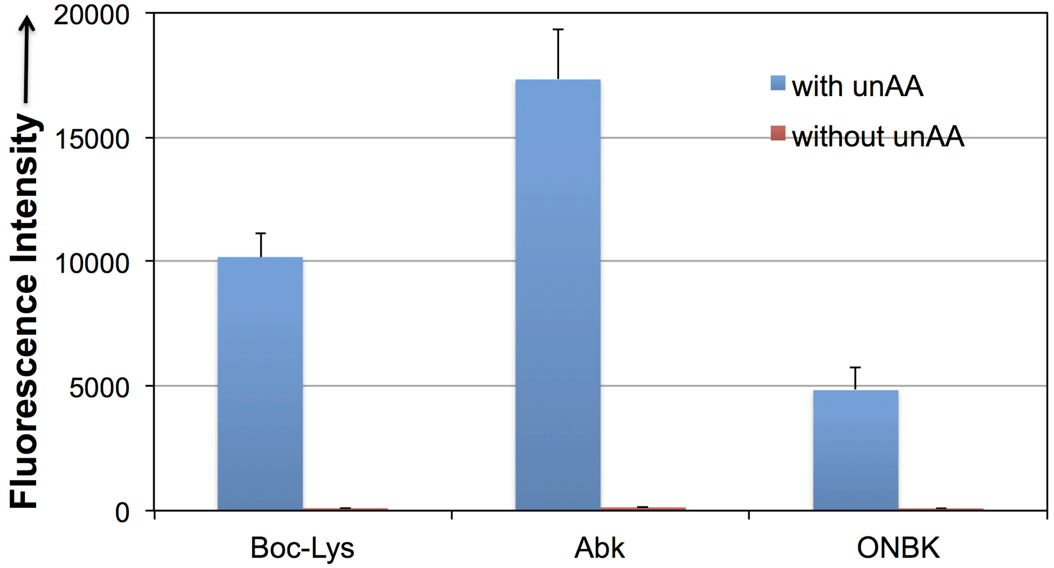

Figure S8. Incorporation of Boc-Lys, Abk, and ONBK in response to amber codon. Fluorescence readings of *E. coli* C321.ΔA cells expressing $tRNA^{Pyl}_{CUA}$, GFP_{UV}-Asn149UAG, and a PylRS variant of interest (BocLysRS, AbkRS, or ONBKRS). The expressions were conducted either in the presence or absence of an unAA of interest (Boc-Lys, 5 mM; ABK, 1 mM; ONBK, 1 mM). Fluorescence intensity was normalized to cell growth. Each data point is the average of triplicate measurements with standard deviation. Boc-Lys, *N*ε-(tert-butyloxy-carbonyl)-L-lysine; AbK, 3'-azibutyl-N-carbamoyl-lysine; ONBK, *o*-nitrobenzyl-oxycarbonyl-Nε-L-lysine.

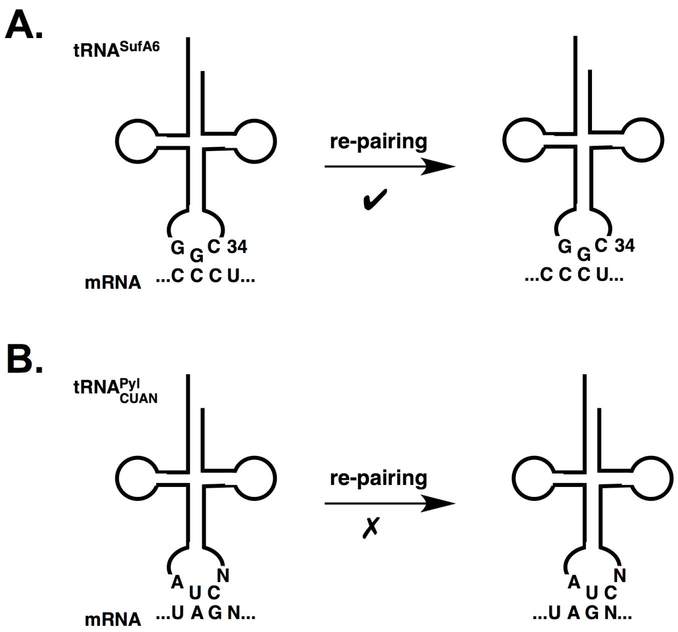

Figure S9. Anticodon-mRNA re-pairing in the P site.

(A) A favorable re-pairing of $tRNA^{SufA6}$ in the +1 frame; (B) An unfavorable re-pairing of $tRNA^{Pyl}_{NCUA}$ in the +1 frame.

Table S1. Additional $tRNA^{Pyl}_{NCUA}$ mutants with improved UAGN decoding activity. Sequences of each $tRNA^{Pyl}_{NCUA}$ variant at randomized positions are listed.

Codon	tRNA variants	Positions				
		29-31	32,33	33.5	37, 38	39-41
UAGA	UAGA-4	C G G	C U	U	A U	C U U
	UAGA-5	G G G	C U	U	A U	C C C
	UAGA-6	G G A	C U	U	A U	C U C
UAGU	UAGU-4	U G G	C U	A	A U	C U U
UAGG	UAGG-4	U G G	C U	A	A U	C U U

Notes:

(1) UAGU-4 AND UAGG-4 are converged into the same sequence.

(2) The tRNA sequences are based on the sequences of the coding DNA.
